# The profile of steroid hormones in human fetal and adult ovaries

**DOI:** 10.1186/s12958-024-01233-7

**Published:** 2024-05-22

**Authors:** Paraskevi Vazakidou, Sara Evangelista, Tianyi Li, Laetitia L. Lecante, Kristine Rosenberg, Jacco Koekkoek, Andres Salumets, Agne Velthut-Meikas, Pauliina Damdimopoulou, Séverine Mazaud-Guittot, Paul A. Fowler, Pim E.G. Leonards, Majorie B.M. van Duursen

**Affiliations:** 1 Section Environment and Health, Amsterdam Institute for Life and Environment, De Boelelaan 1085, Amsterdam, 1081 HV The Netherlands; 2 Department of Gynecology and Reproductive Medicine, Karolinska University Hospital, Stockholm, SE-14186 Sweden; 3 Division of Obstetrics and Gynecology, Department of Clinical Science, Intervention and Technology, Karolinska Institutet, Stockholm, SE-14186 Sweden; 4 Institute of Medical Sciences, School of Medicine, Medical Sciences & Nutrition, University of Aberdeen, Foresterhill, Aberdeen, AB25 2ZD UK; 5 Department of Chemistry and Biotechnology, Tallinn University of Technology, Tallinn, Estonia; 6 Nova Vita Clinic, Tallinn, Estonia; 7 Competence Center on Health Technologies, Tartu, Estonia; 8 Department of Obstetrics and Gynaecology, Institute of Clinical Medicine, University of Tartu, Tartu, Estonia; 9 Univ Rennes, EHESP, Irset (Institut de recherche en santé, environnement et travail) – UMR_S 1085, Inserm, Rennes, F-35000 France

**Keywords:** Fetal and adult ovarian steroidogenesis, Backdoor pathway, Corticosteroids, 17 alpha estradiol

## Abstract

**Background:**

Reproduction in women is at risk due to exposure to chemicals that can disrupt the endocrine system during different windows of sensitivity throughout life. Steroid hormone levels are fundamental for the normal development and function of the human reproductive system, including the ovary. This study aims to elucidate steroidogenesis at different life-stages in human ovaries.

**Methods:**

We have developed a sensitive and specific LC-MS/MS method for 21 important steroid hormones and measured them at different life stages: in media from cultures of human fetal ovaries collected from elective terminations of normally progressing pregnancy and in media from adult ovaries from Caesarean section patients, and follicular fluid from women undergoing infertility treatment. Statistically significant differences in steroid hormone levels and their ratios were calculated with parametric tests. Principal component analysis (PCA) was applied to explore clustering of the ovarian-derived steroidogenic profiles.

**Results:**

Comparison of the 21 steroid hormones revealed clear differences between the various ovarian-derived steroid profiles. Interestingly, we found biosynthesis of both canonical and “backdoor” pathway steroid hormones and corticosteroids in first and second trimester fetal and adult ovarian tissue cultures. 17α-estradiol, a less potent naturally occurring isomer of 17β-estradiol, was detected only in follicular fluid. PCA of the ovarian-derived profiles revealed clusters from: adult ovarian tissue cultures with relatively high levels of androgens; first trimester and second trimester fetal ovarian tissue cultures with relatively low estrogen levels; follicular fluid with the lowest androgens, but highest corticosteroid, progestogen and estradiol levels. Furthermore, ratios of specific steroid hormones showed higher estradiol/ testosterone and estrone/androstenedione (indicating higher CYP19A1 activity, *p* < 0.01) and higher 17-hydroxyprogesterone/progesterone and dehydroepiandrosterone /androstenedione (indicating higher CYP17A1 activity, *p* < 0.01) in fetal compared to adult ovarian tissue cultures.

**Conclusions:**

Human ovaries demonstrate de novo synthesis of non-canonical and “backdoor” pathway steroid hormones and corticosteroids. Elucidating the steroid profiles in human ovaries improves our understanding of physiological, life-stage dependent, steroidogenic capacity of ovaries and will inform mechanistic studies to identify endocrine disrupting chemicals that affect female reproduction.

**Supplementary Information:**

The online version contains supplementary material available at 10.1186/s12958-024-01233-7.

## Background

Approximately 200 million tons of synthetic chemicals are released to the market annually, which may endanger female reproductive health in humans and wildlife (WHO-UNEP, 2013). Specifically, chemicals that target the endocrine system, i.e. endocrine disrupting chemicals (EDCs), have the potential to affect the female reproductive system via different modes of action [1]. Severe reproductive problems in the daughters born to women who had taken the synthetic estrogen diethylstilbestrol (DES) during pregnancy [2], clearly shows that the developing human female reproductive tract is also sensitive to endocrine disruption.

A complex network of steroid hormone synthesis, action and regulation is crucial for human ovarian development and physiology. During early human embryonic development, primordial germ cells migrate to the primitive gonad, form mitotic germ cell nests at gestational week (GW) 10, and ovarian differentiation begins. Follicle assembly and primordial follicle formation is initiated around GW 17 [1, 3]. At birth, each ovary contains around three hundred thousand primordial follicles. This is the ovarian reserve, which gives rise to a sharply finite number of ovulations in adult life [4–6]. In contrast to the male, ovarian development has been considered less hormonally dependent since complete absence of androgens and/or androgen signalling leads to a superficially female phenotype [7]. However, we have shown that the human fetal ovary expresses high levels of estrogen receptor beta and has steroid hormone synthesis capacity [8, 9], with pre-granulosa cells in primordial follicles expressing abundant CYP19A1. This indicates an important role for steroid hormones in ovarian development and function. While the specific steroid hormones present at different life stages and their roles in ovarian development remain poorly understood [10, 11], disruption of ovarian development by chemical exposure may lead to reproductive disorders later in life, known as ovarian dysgenesis syndrome [1, 12]. In adulthood, recruited primordial follicles start forming primary, secondary, antral follicles and pre-ovulatory follicles, a maturation process known as folliculogenesis [13]. Follicular theca and granulosa cells support the development and maturation of the oocyte via steroid hormone production. During each ovarian cycle in women, antral follicles mature with only one dominant follicle selected for ovulation, while others undergo apoptotic atresia. After ovulation, the follicle becomes the corpus luteum, producing hormones such as high levels of progesterone. EDCs can interfere with the ovarian cycle and oocyte maturation. For example, there are associations between phthalate exposure and disrupted follicular growth, gonadotropin and hormone levels in rodents and cows, as well as reproductive complications in humans [14]. Other studies have associated persistent organic pollutants (POPs) and di-2-ethylhexyl phthalate (DEHP) metabolites in follicular fluid with poor reproductive outcomes in women receiving infertility treatment [15, 16].

**Fig. 1:**
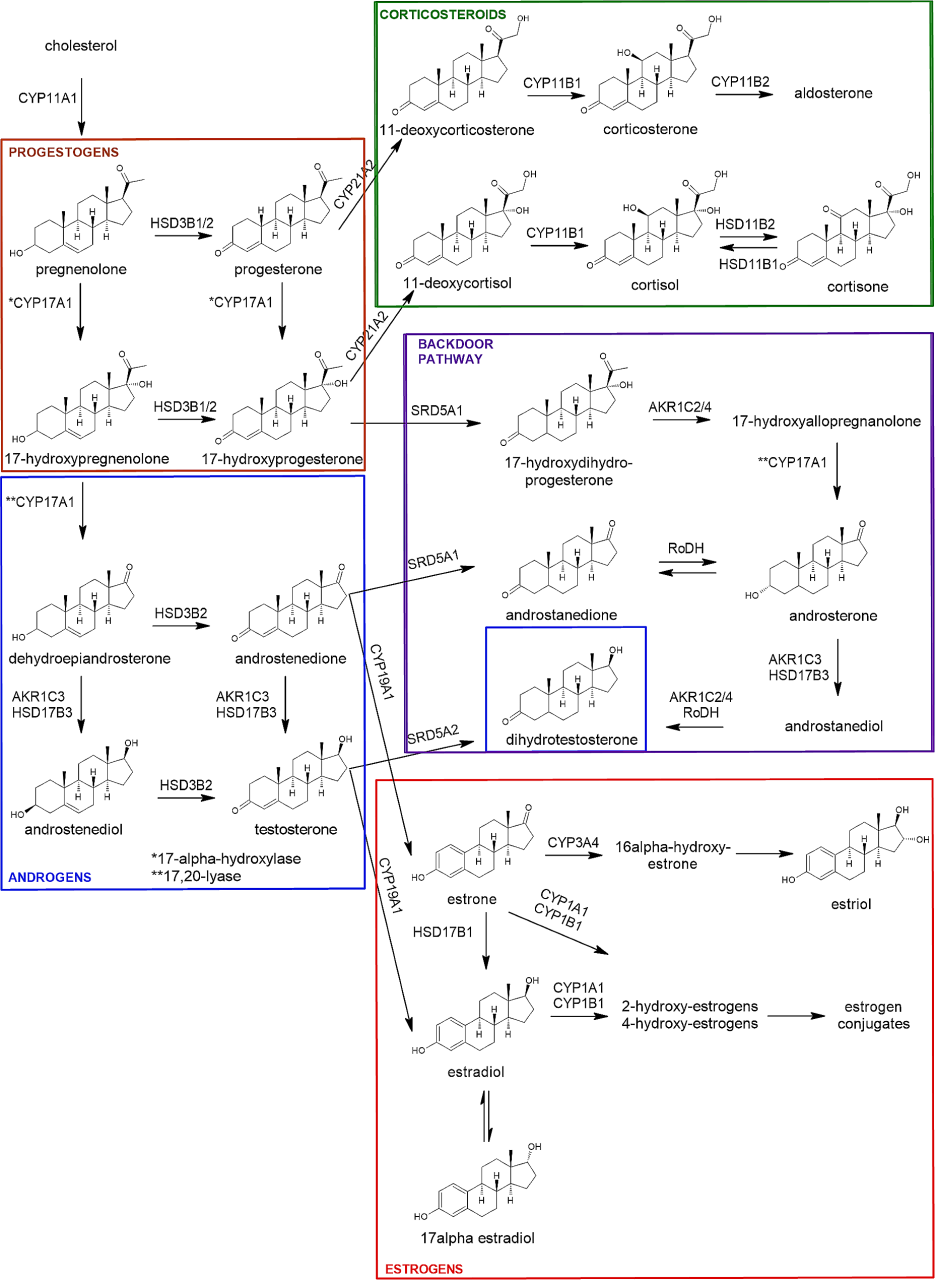
Simplified steroidogenic pathway. Steroid hormones shown together with their structures represent those measured in the present study. Progestogens and androgens from the canonical pathway (brown and blue boxes, respectively) and from the non-canonical (backdoor) pathway (purple box) are presented. Estrogens (red box) and corticosteroids (green box) are shown. The enzymes involved are given above the arrows. CYP11A1 (cholesterol side-chain cleavage enzyme, P450scc), CYP17A1 (17α-hydroxylase/17,20-lyase), HSD3B1/2 (3β-hydroxysteroid dehydrogenase type 1/2), HSD17B1/3 (17β-hydroxysteroid dehydrogenase type 1/3), SRD5A1/2 (5α-reductase type 1/2), AKR1C2/3/4 (aldo-keto reductase family 1 member C2/C3/C4), RoDH (retinol dehydrogenase/17β-hydroxysteroid dehydrogenase type 6), CYP21A2 (21α-hydroxylase type 2), CYP11B1/2 (11β-hydroxylase type 1/2), HSD11B1/2 (11β-dehydrogenase type 1/2), CYP19A1 (aromatase), CYP1A1 (cytochrome P450 1A1), CYP1B1 (cytochrome P450 1B1), CYP1B2 (cytochrome P450 1B2), CYP3A4 (cytochrome P450 3A4)

Steroidogenesis, steroid hormone biosynthesis (Fig. 1), involves several enzymatic conversions of the precursor cholesterol [13]. Luteinizing hormone (LH) regulates the production of progestogens and androgens, mainly androstenedione, in theca cells using cholesterol derived from lipoproteins in blood. Follicular stimulating hormone (FSH) regulates the conversion of androgens androstenedione and testosterone, into estrogens, estrone and estradiol, in granulosa cells. Dihydrotestosterone is the most potent androgen produced after multiple conversions of progestogens and androgens by enzymes from the well-known classic steroidogenic pathway, however, more recently an alternative steroidogenic pathway has been described. This alternative or “backdoor” steroidogenic pathway differentiates at the level of 17-hydroxyprogesterone from the classic canonical pathway and ultimately leads to biosynthesis of dihydrotestosterone through other non-classic steroid intermediates, thereby by-passing testosterone [17, 18]. Enzymes from the backdoor pathway are also expressed in human ovarian theca cells, and protein expression of this pathway is increased in ovaries from women with polycystic ovary syndrome, PCOS [19].

Understanding steroidogenic profiles in human ovarian tissues is crucial to improve regulation and testing strategies for identification of EDCs that target steroid hormone biosynthesis [20]. While ovarian development and function are highly hormonally regulated, and EDCs are known to affect these processes, the effect of EDCs on ovarian steroidogenesis and, subsequently, on female fertility remain poorly understood. This is partly due to the lack of human data on differences in steroid hormone profiles throughout a woman’s life. To this end, we have developed an analytical method to simultaneously quantify 21 steroids (Fig. 1) in primary human ovarian-derived samples. Comparison of the steroid profiles in these ovarian-derived samples demonstrated clear differences at different life stages from fetal to adult.

## Materials and methods

### Chemicals and materials

Cortisol, cortisone, corticosterone, 11-deoxycortisol, 11-deoxycorticosterone, androstenedione, dehydroepiandrosterone, testosterone, dihydrotestosterone, 5α-androsterone, 17α-hydroxyprogesterone, progesterone, pregnenolone, 17α-hydroxypregnenolone, 17β-estradiol, 17α-estradiol, estrone, estriol were purchased from Sigma-Aldrich. 5α-Androstenediol was acquired from Steraloids and 5α-Androstanedione from Eurisotop. 17α-hydroxydihydroprogesterone was purchased from Santa-Cruz. All native steroids had more than 99% purity. In addition, internal standards cortisol-9,11,12,12-D4, corticosterone-9,11,12,12-D4, 11-deoxycortisol-2,2,4,6,6-D5, progesterone-2,3,4-13C3, androstene-3,17-dione-2,3,4-13C3, 5α-dihydrotestosterone-16,16,17-D3, estrone-2,3,4-13C3, 17β-estradiol-2,3,4-13C3 were purchased from Sigma-Aldrich. Moreover, the internal standards pregnenolone-20,21-13C2-16,16-D2, 5α-androstanedione-2,3,4-13C3, dehydroepiandrosterone-2,2,3,4,4,6-D6, testosterone-2,3,4-13C3, estriol-13C3 were purchased from IsoSciences. Cortisone-2,2,4,6,6,9,12,12-D8, 17α-hydroxyprogesterone-2,3,4-13C3, 5α-androsterone-2,2,4,4-D4 from Eurisotop and 5α-androstenediol-16,16,17-D3 from Steraloids. Purity of all internal standards were more than 98%. Steroid names, abbreviations and CAS numbers are provided in Supplementary Table S1 and Table S2.

Solvents and reagents used for the steroid analysis were methanol (Biosolve BV), Milli-Q water (Millipak, Merck), acetone (Biosolve BV), formic acid (99%, Biosolve Chimie), sodium bicarbonate and sodium carbonate (Sigma-Aldrich), dansyl chloride (Sigma-Aldrich) and ammonium fluoride (Sigma-Aldrich). Cell media used for the validation of the analytical method consisted of DMEM/F-12 medium w/o phenol red (REF no. 11039-021), ITS + premix (cat. 354,352), Serum Charcoal dextran treated Hyclone (SH.30068.03), 1% Penicillin Streptomycin (REF no. 15140-122) and 0.1% dimethylsulfoxide (DMSO).

### Human fetal ovarian cultures

For steroid quantification, 8 fetal ovaries were collected from first trimester fetuses between GW 10 (10 weeks of gestation) and GW 14 + 1 (14 weeks and 1 day of gestation) and another 7 fetal ovarian tissues from second trimester fetuses between GW 15 + 2 and 19 + 5 (Table S3). Fetal material was obtained as a part of the Scottish Advanced Fetal Research (SAFeR) Study as approved by NHS Grampian Research Ethics Committees (REC 15/NS/0123). Women over 16 years of age and between 7 and 20 gestational weeks (GW) seeking elective terminations of normally progressing pregnancy (determined at ultrasound scan prior to termination) were recruited with full written, informed consent by NHS Grampian research nurses working independently of the study. The aim of the study was to study the effects of EDCs on fetal ovaries. Fetuses were collected following termination by RU486 (mifepristone) treatment (200 mg) and misoprostol-induced delivery as previously described [21]. Fetal tissues were transported to the laboratory within 30 min of delivery, weighed, sexed and the crown-rump length as well as the foot length were recorded for further confirmation of gestational age. Ovaries separated from mesonephroi were dissected, weighed, and immediately placed into ice-cold phosphate-buffered saline. Fetal ovaries were processed as previously described [10]. Briefly, each ovary was cut into 1 mm^3^ size explants to ensure accessibility to nutrients and viability of the tissue. The same number of ovarian explants randomly picked from each ovary of the same fetus (2 per ovary, 4 in total) was used for each culture condition. Explants were placed into cell culture inserts (0.4 μm pores, Millipore #PICM01250) in wells filled with 400 µl of culture media containing 0.1% v/v dimethyl sulfoxide (DMSO). Culture media included phenol red-free Medium 199 (Invitrogen Life Technologies, Cergy-Pontoise, France) supplemented with 50 µg/mL gentamycin, 2.5 µg/mL fungizone (Sigma Aldrich Chemicals, Saint-Quentin Fallavier, France), and 1 g/L insulin, 0.55 g/L transferrin, and 0.67 mg/lsodium selenite (ITS, Corning, Fisher Scientific). Cultures were incubated for 7 days at 37 °C under 5% CO_2_ and humidity. Viability of tissue explants in culture was confirmed with immunohistochemistry using anti-cleaved caspase 3 antibody. Media were completely changed at first after 24 h and then every 48 h. Finally, culture media were collected, snap-frozen and stored at − 80 °C until steroid hormone analysis. Clinical metadata regarding the maternal age, BMI and smoking status according to questionnaire are given in Supplementary Table S3.

### Human adult ovarian cultures

For this study, 13 human adult ovary culture-conditioned media samples were collected for steroid quantification. Human adult ovaries were collected from women undergoing Caesarean section (C-section), as described by Li et al. and part of the steroid hormone analysis data for these samples is re-used in the present study [22]. All women were younger than 35 years and gave consent to participate in the research project investigating the effects of EDCs on ovaries at Karolinska University Hospital Huddinge in Stockholm, Sweden, in accordance with the Declaration of Helsinki. Briefly, a superficial piece of cortical tissue (5 mm * 5 mm * 2 mm depth) was taken, placed in Dulbecco’s phosphate buffered saline with calcium, magnesium, glucose, sodium pyruvate (Life Technologies, Paisley, UK) and transferred to the laboratory within 10 min. Upon arrival in the laboratory, ovarian cortical tissues were cut into 1 mm * 1 mm pieces using scalpels and each piece was placed on a laminin-221 coated Millicell cell culture plate insert (Merck Millipore, Darmstadt, Germany) in 24-well plates (Sarstedt, Nümbrecht, Germany). The tissues were cultured in an incubator at 37 °C under 5% CO_2_. Full media change (350 µL) with 0.1% DMSO was performed every other day. Full culture media contained phenol red high glucose DMEM with glutamax (Life Technologies Grand Island, NY, USA) supplemented with 10% human serum albumin solution (Vitrolife, Göteborg, Sweden), 1% antibiotic-antimycotic (Life Technologies, Grand Island, NY, USA), 1% insulin-transferrin-selenium (Life Technologies, Grand Island, NY, USA), and 0.5 IU/ml human recombinant follicle-stimulating hormone (FSH, Fostimon, Italy). After 6 days of culture, media from 2 technical replicates were pooled and stored at -80 °C until steroid hormone analysis. LDH-Glo cytotoxicity assay kit (Promega, USA) was used to assess cytotoxicity for the ovarian tissue in culture. Clinical metadata regarding the women’s age, BMI, smoking status, disease and androgen treatment are given in Supplementary Table S4.

### Human adult follicular fluid

Follicular fluids were collected from women undergoing infertility treatment in Estonia, as described by Bellavia et al. [15]. The full cohort encompassed 148 women (age: 23–43) undergoing fertility treatment at Nova Vita Clinic in Tallinn, and 14 follicular fluid samples were randomly selected for the present study. All women were informed regarding the study, and they signed an informed written consent form complying with the Declaration of Helsinki. Briefly, the follicular fluid samples were collected from the leading follicles avoiding blood contamination, centrifuged for 10 min at 300 g and then for another 10 min at 2000 g [15]. To prevent dilution of the samples, the flushing medium was eliminated from the needle and the hose prior to collection of follicular fluid. The cell-free follicular fluid samples after centrifugation were aliquoted, and within 2 h delivered on ice to the university where the samples were kept frozen at -80 °C till steroid analysis. Clinical metadata of the women in this study are given in Supplementary Table S5.

### Quantification of steroid hormones

The samples were thawed at room temperature and extracted with offline solid phase extraction (SPE). 100 µl of each sample and 50 µl internal standard were added to 1 ml Milli-Q (Millipak, Merck) with 2% formic acid (Sigma-Aldrich), which made 1.15 ml loading solution for each sample ready for the SPE. The SPE was conducted with Agilent Versaplate-Plexa 96 well-plate preassembled with Bond Elut Plexa 30 mg cartridges. First, the cartridges were pre-conditioned with 0.5% formic acid in 1 ml methanol (Biosolve BV). Then, the cartridges were equilibrated with 0.5% formic acid in 1 ml Milli-Q and each sample was loaded afterwards. After this, the cartridges were pre-washed with 1 ml Milli-Q and washed by 1 ml methanol/Milli-Q (30:70). Vacuum was applied for 10 min. Finally, the steroid hormones were eluted with 0.7 ml methanol and collected to a 96 well-plate and vacuum was briefly applied to complete elution. Subsequently, the extracts were evaporated to dryness at 40 °C using CentriVap Concentrator (LABCONCO), were reconstituted with 150 µl methanol/Milli-Q (50:50) and were filtrated with Agilent Captiva 96 well filter plate (0.2 μm). Then the samples were analysed using LC-MS/MS for quantifying underivatised free steroids.

After analysing the underivatised steroid hormones, the samples were then used for quantifying low amounts of estrogens after dansylation. The samples were evaporated to dryness at 40° C using CentriVap Concentrator (LABCONCO) and subsequently 40 µl bicarbonate buffer (pH 10.5) and 40 µl dansyl chloride (Sigma-Aldrich) (1 mg powder in 1 ml acetone (Biosolve BV)) were added. The reaction was completed in the oven for 5 min at 60 °C. Then the samples were ready for analysis of the dansylated estrogens.

Steroid levels (pg/ml) found in procedural blanks (SPE extracted blank media) were subtracted. Steroid levels (pg/ml) found in blank media samples were subtracted from adult ovarian tissue cultures. No blank media samples were available from follicular fluid samples. While, only cortisol (CO-SOL) was quantified in higher level in the blank media than the levels in the fetal ovarian tissue samples, which was excluded from the measurements of fetal samples as explained in Results.

Both derivatised and underivatised samples were analysed with LC-MS/MS using AB Sciex 6500 + triple quadrupole mass spectrometer coupled to an AB Sciex Exion LC system. Ionisation was achieved with electrospray ionisation (ESI) in positive mode and only for underivatized estrogens in negative mode. The column used was Phenomenex Kinetex C18 column (150 × 3 mm, 2.6 μm particle size, pore-size 100 Å) with mobile phases A: Milli-Q water with 0.2 mM ammonium fluoride (Sigma-Aldrich) and B: methanol. The flow rate was 0.6 ml/min and the injection volume was 10 µl. The analytical method was validated in cell media spiked with steroid standards.

### Steroid hormones validation

The method was validated for underivatized steroid hormones based on one level (6 ng/ml) for inter-day repeatability and based on two levels (300 pg/ml and 3 ng/ml) with spiked cell media for assessing intra-day repeatability. For the inter-day repeatability at 6 ng/ml, the accuracy was on average 90% (64-109%) and precision was 15% (4.2-57%) for all underivatised steroids. Similarly, dansylated estrogens were assessed for inter-day repeatability at 10 pg/ml. Accuracy and precision for all dansylated estrogens were on average 84% (79-92%) and 18% (14-22%) respectively. More information about the intra-day and inter-day repeatability assessment, limit of detection, transitions and calibration range is provided in Supplementary Tables S6-S8.

### Statistical analysis

Steroid hormone peak data were processed with Sciex Analyst 1.7.2 software and further calculations were completed with Excel (Version 2208 Build 16.0.15601.20540). Steroid hormone concentrations for fetal and adult ovarian tissue cultures were normalised based on the volume of the culture media collected, size and number of tissue explants and time of incubation, based on which the corresponding steroid hormone release rates were calculated.

Graphs with steroid hormone release rates or concentrations and corresponding ratios were made with GraphPad Prism 9.5.0, and principal component analysis (PCA) was performed in R version 4.3.0 using base and attached packages: ggrepel_0.9.3, factoextra_1.0.7 ggplot2_3.4.2, FactoMineR_2.8 in R studio (version 2023.03.1 + 446) after data normalization in MetaboAnalyst 5.0. For the PCA plot, probabilistic quotient normalization, logarithmic transformation, and pareto scaling were used. Missing values were replaced by 1/5 of lowest release rate and steroid hormones with more than 55% missing values were eliminated, leading to a final dataset of only 13% predicted missing values. Logarithmic transformation of steroid hormone release rates or ratios were normally distributed, based on Shapiro-Wilk test and quantile-quantile plots. Unpaired multiple t-test assuming equal standard deviation using Holm-Šídák method and p value threshold set to 0.05 was applied for each steroid hormone release rate or ratio coming from two different sample types: first compared to second trimester fetal ovarian tissue cultures; first trimester fetal ovarian tissue cultures compared to adult ovarian tissue cultures; second trimester fetal compared to adult ovarian tissue cultures; adult ovarian tissue cultures compared to follicular fluid. Comparisons were performed between means of steroid hormone release rates and ratios. The units for steroid hormones were different in adult ovarian tissue cultures and follicular fluid and thus only steroid hormone ratios were compared in this case. Outlier detection for adult ovarian tissue cultures was performed with Rout and with Grubb’s tests and was confirmed visually with principal component analysis.

## Results

### Steroid hormone profile in fetal ovarian tissue cultures

Progestogens such as pregnenolone (P5), 17α-hydroxyprogesterone (17OH-PROG), progesterone (P4) exhibited the highest release rates of steroid hormones among all fetal ovarian tissue cultures from first (*N* = 8) and second trimester (*N* = 7) (Table S9; Fig. S1). Dehydroepiandrosterone (DHEA) had a high release rate in several fetal ovarian tissue cultures (first trimester: *N* = 3, second trimester: *N* = 6) (Fig. S1; Table S9). In contrast, 11-deoxycorticosterone (11-DOC), 11-deoxycortisol (11-DOS), androstenedione (A4) and estrone (E1) had low release rates in almost all fetal ovarian tissue cultures (Table S9; Fig. S1). Cortisol (CO-SOL) was also detected in fetal ovarian tissue cultures (Table S10). However, CO-SOL was also quantified in culture media blank, even in higher levels than in fetal ovarian tissue culture samples (Table S10). Therefore, CO-SOL was excluded from analysis.

**Fig. 2:**
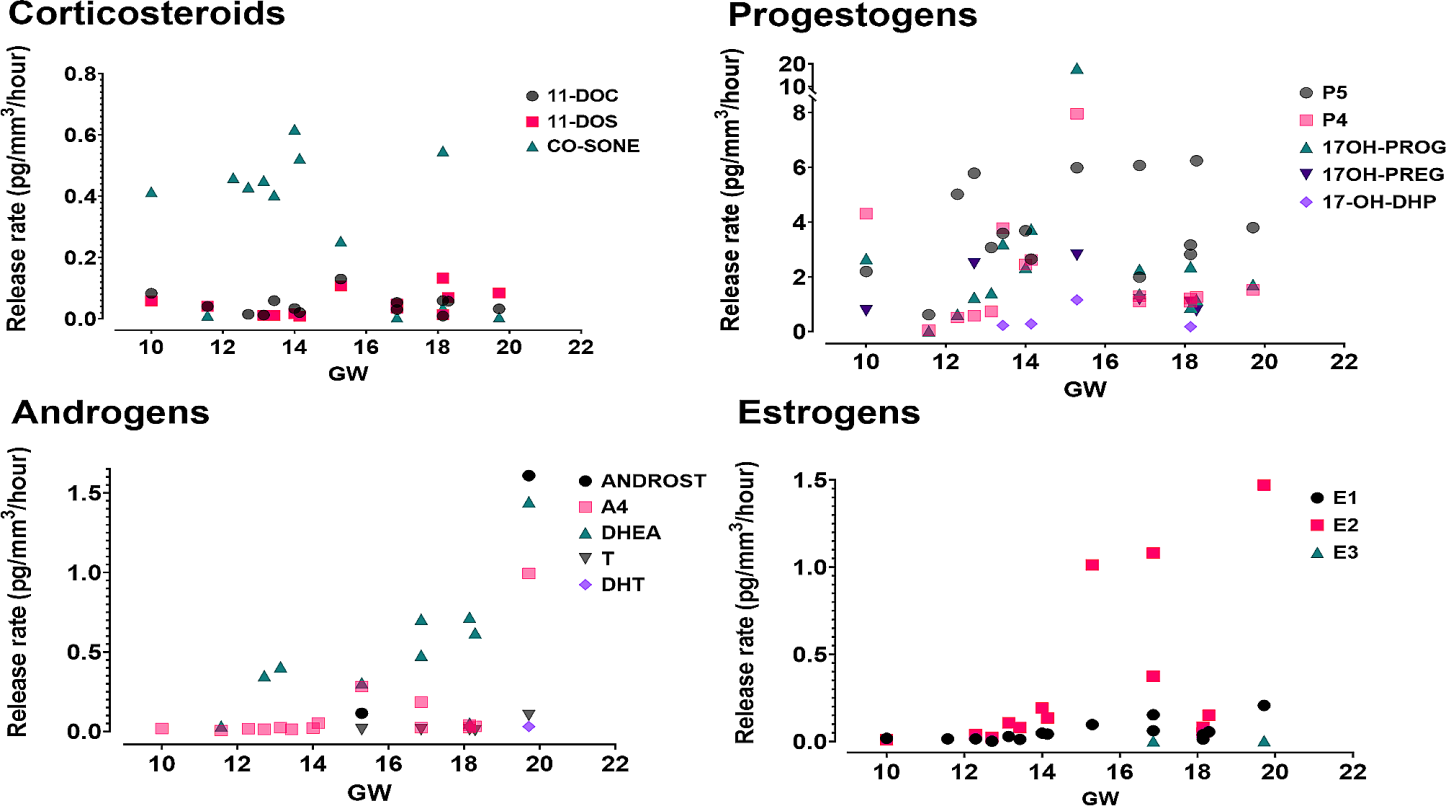
Steroid hormone release rates in human fetal ovarian tissue cultures at different fetal ages (GW, gestational week). Each coloured symbol represents a specific steroid hormone release rate (pg/mm^3^/hour)

Release rates of all steroid hormones measured in culture media of first (*N* = 8) and second trimester (*N* = 7) fetal ovarian tissues showed high inter-individual variability (Fig. 2). Samples from fetuses older than GW14, i.e., of the second trimester (Table S3), showed generally higher levels of estrogens and androgens, compared to the first trimester fetal samples (Fig. 2). A4, E1 and estradiol (E2) release rates were statistically significantly higher in samples from the second trimester than in those from the first trimester (Table S9; Fig. S1).

In contrast, T, DHT and E3 were detected only in few samples of the second trimester, with the lowest release rates (T: *N* = 5, DHT: *N* = 1, E3: *N* = 2) (Table S9; Fig. S1). In addition, two backdoor pathway steroids were detected in fetal samples: 17α-hydroxydihydroprogesterone (17-OH-DHP) was detected in two samples of each trimester, while 5α-androsterone (ANDROST) was detected in only two second trimester samples (Table S11; Fig. S1).

### Steroid hormone profile in adult ovarian cortex tissue cultures

The release rate of P5 was the highest of all release rates measured in adult ovarian cortex tissue cultures (*N* = 13), followed by DHEA, 17-hydroxypregnenolone (17OH-PREG), E2, 17-OH-PROG, A4 and T (Table S9; Fig. S2). Estrogens and progestogens as well as A4, T, CO-SONE and CO-SOL showed considerable inter-individual variability (Table S9; Fig. S2). Corticosteroids, DHT and E3 had the lowest release rates of all the hormones analysed (Table S9; Fig. S2). Backdoor pathway steroid hormones 17-OH-DHP, ANDROST and 5α-androstanedione (AND-AN), were found only in one out of 13 samples (Table S11). Notably, this sample had the highest release rate of almost all steroid hormones measured, including all estrogens (Fig. S2). This sample was statistically an outlier and thus was excluded from further comparisons.

### Steroid hormone profiles in fetal and adult ovarian tissue cultures

**Fig. 3:**
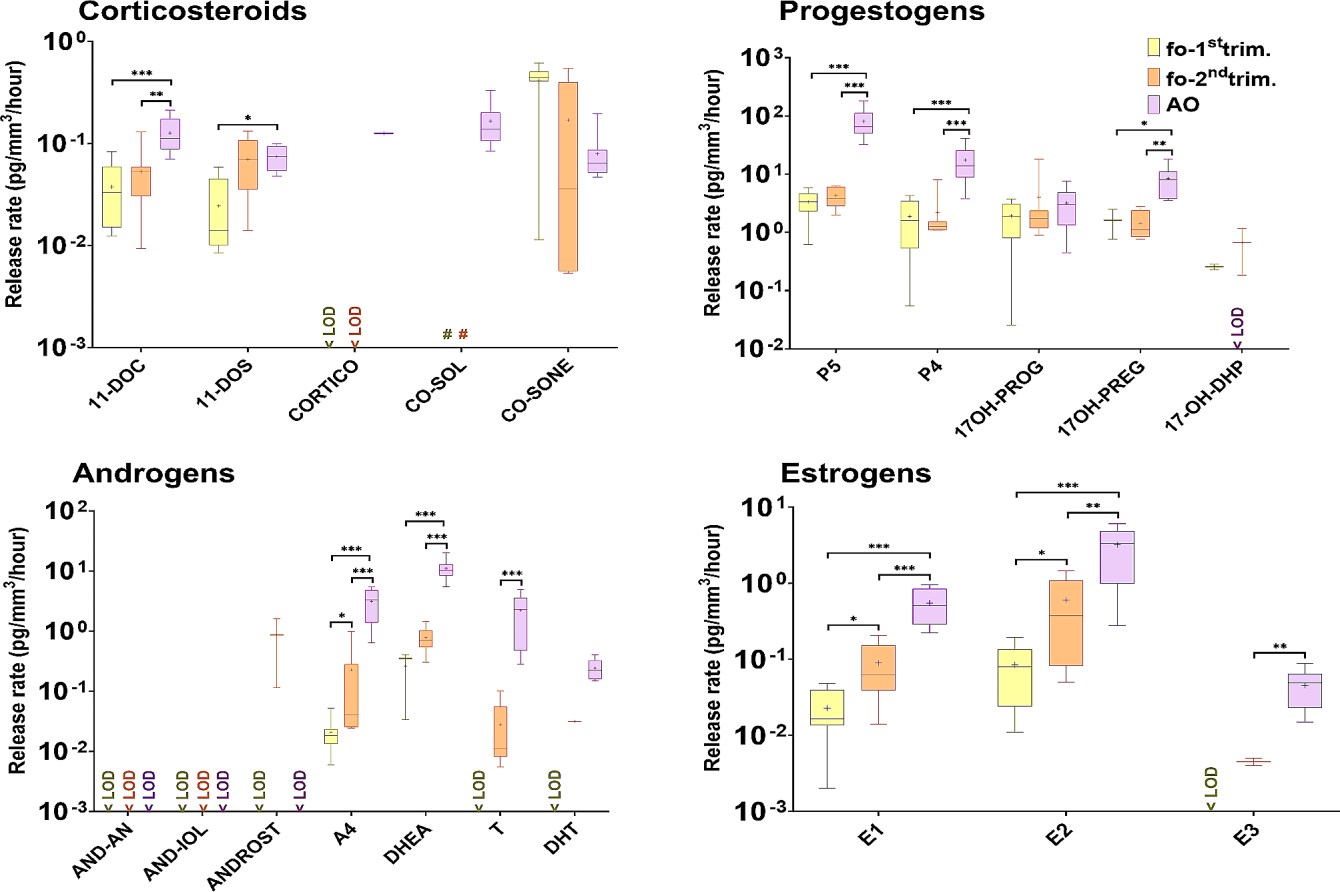
Life stage differences in the profiles of steroid hormone release rates from first (fo-1st trim.) and second (fo-2nd trim.) trimester fetal ovarian tissue cultures and adult (AO) ovarian tissue cultures. Release rates (pg/mm^3^/ hour) are shown on a logarithmic scale on the Y-axis. Median release rates (middle line), 25% and 75% percentiles (box borders) are shown. Mean release rates are indicated as ”+” and whiskers indicate the minimum and maximum values. N values are given in Figure S1. Statistically significant differences in steroid hormone release rates between fetal and adult ovarian cultures are shown by *(*p* < 0.05), **(*p* < 0.01) and ***(*p* < 0.001). < LOD, below the limit of detection.”#” used for levels of CO-SOL measured in fetal ovarian tissue cultures, which were excluded since CO-SOL was measured in the uncultured media (See in Results)

Steroid hormone profiles from first (*N* = 8) and second trimester (*N* = 7) fetal ovarian tissue cultures and adult ovarian tissue cultures (*N* = 12) were compared to examine life-stage differences in ovarian steroidogenic capacity. Almost all measured release rates of progestogens, androgens and estrogens were statistically significantly higher in adult ovarian tissue cultures than in fetal ovarian tissue cultures (Table S9; Fig. 3).

**Fig. 4:**
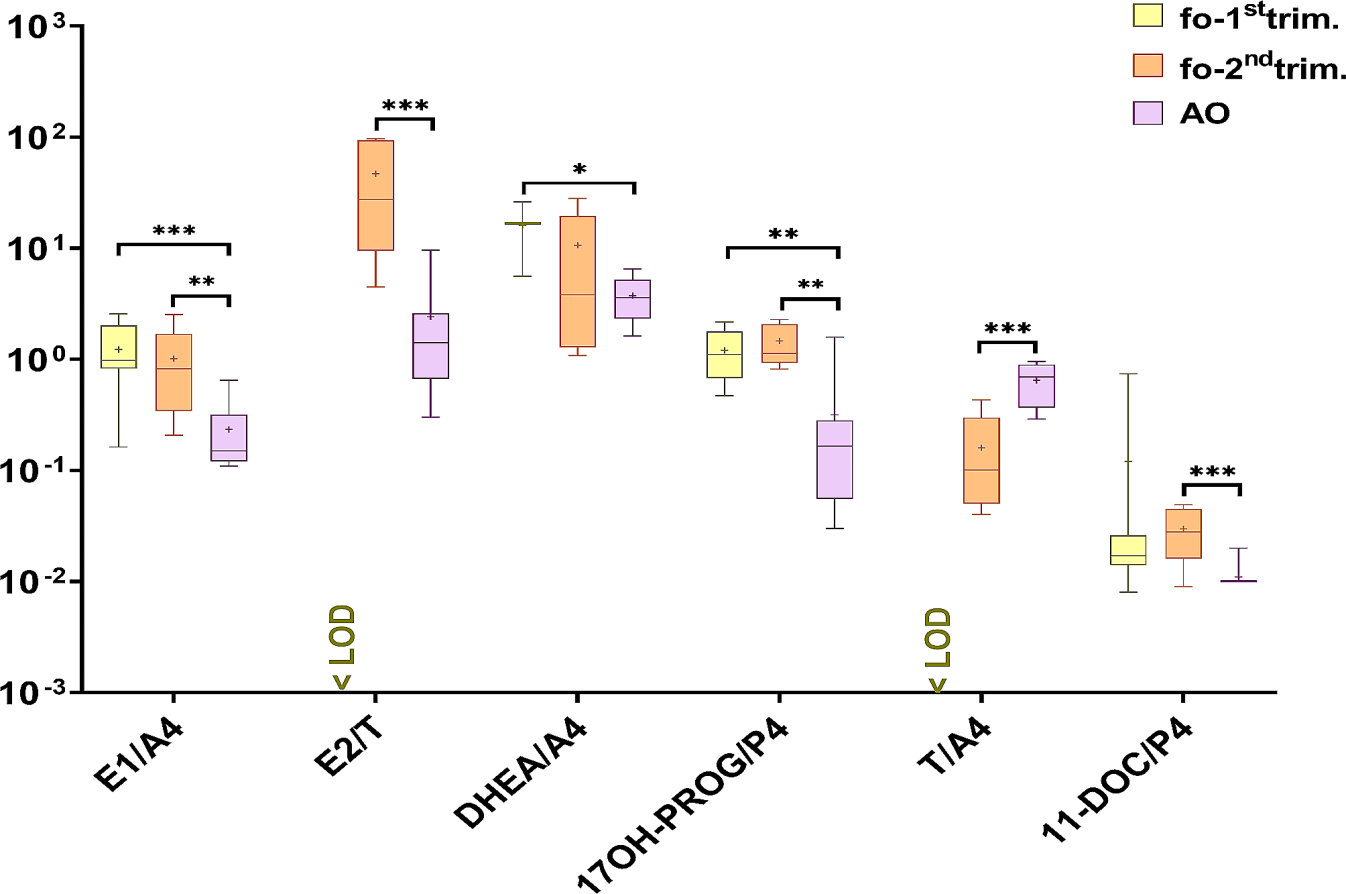
Ratios of steroid hormone release rates from first (fo-1st trim.) and second (fo-2nd trim.) trimester fetal ovarian tissue cultures and adult (AO) ovarian tissue cultures. Median ratios (middle line), 25% and 75% percentiles (box borders) are shown on a logarithmic scale. Mean ratios are indicated as ”+” and whiskers indicate the minimum and maximum values. Statistically significant differences in steroid hormone ratios between follicular fluid and adult ovarian cultures are shown by **(*p* < 0.01) and ***(*p* < 0.001)

Next, steroid hormone product to precursor ratios were calculated to reflect steroidogenic enzymes activity (Table S9; Table S12). The ratios for E1/A4, E2/T, DHEA/A4, 17OH-PROG/P4, 11-DOC/P4 were statistically significantly higher in fetal compared to adult ovarian tissue cultures (Fig. 4). In contrast, the ratio T/A4 was statistically significantly higher in adult samples (Fig. 4).

### Steroid hormone profile in follicular fluid

**Fig. 5:**
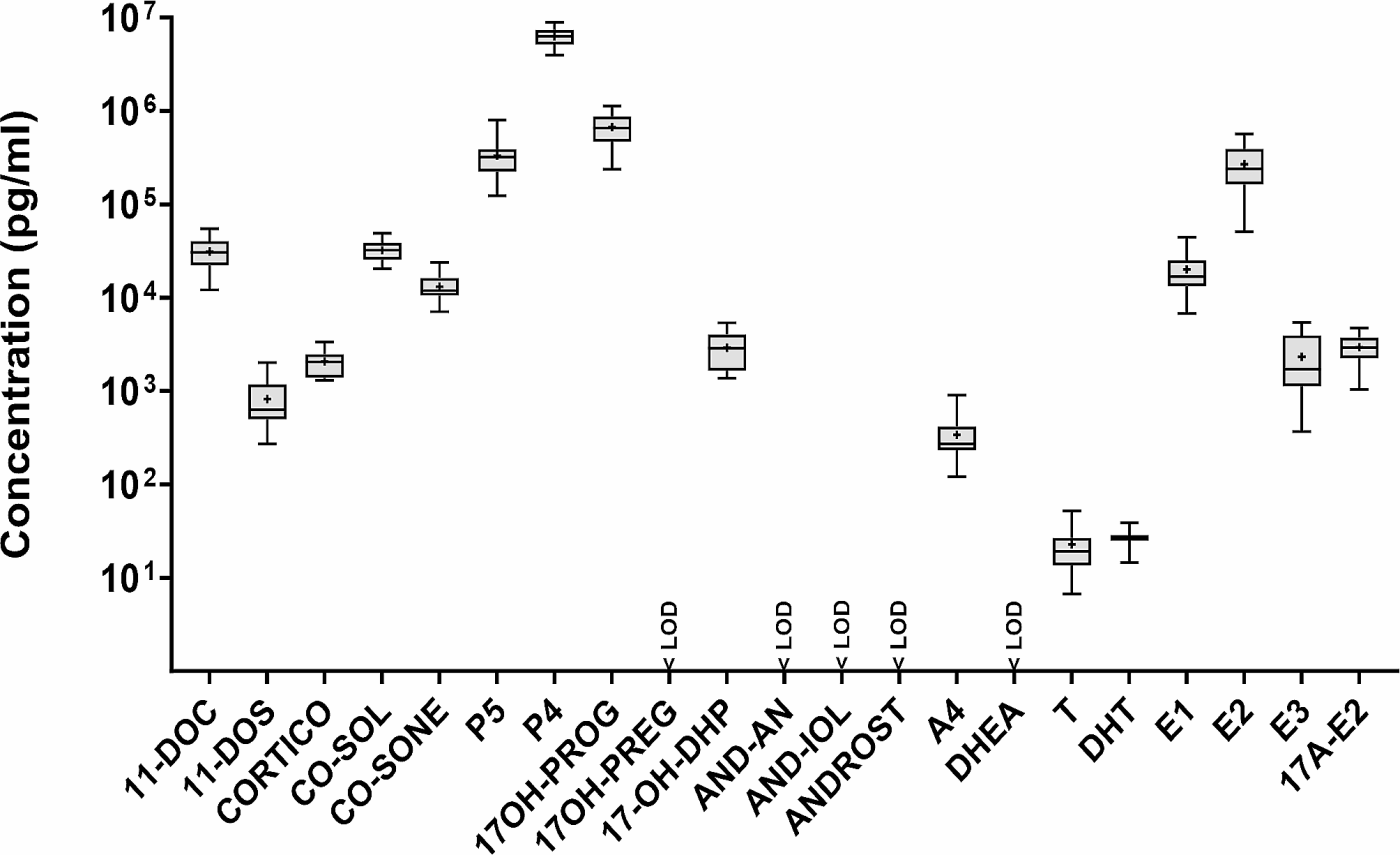
Steroid hormone concentrations (pg/ml) in 14 human follicular fluid samples (FF). Median concentrations (middle line), 25% and 75% percentiles (box borders) are shown on a logarithmic scale. Mean concentrations are indicated as ”+” and whiskers indicate the minimum and maximum values. LOD, limit of detection

Concentrations of progestogens, estrogens and corticosteroids were the highest of all steroid hormones measured in adult follicular fluid samples (*N* = 14) (Fig. 5). Specifically, steroid hormones with the highest concentrations were P4, 17OH-PROG, P5 and E2 (Table S9; Fig. 5). In contrast, steroid hormones with the lowest concentrations were A4 and T, which were found in all samples, and DHT, which was found in only 3 samples (Table S3; Fig. S3). One backdoor pathway steroid hormone, 17-OH-DHP, was detected in 13 of the 14 samples (Fig. S3). Furthermore, 17α-estradiol (17 A-E2) was found in all follicular fluid samples (Fig. S3).

### Adult ovarian steroid hormone profiles

**Fig. 6:**
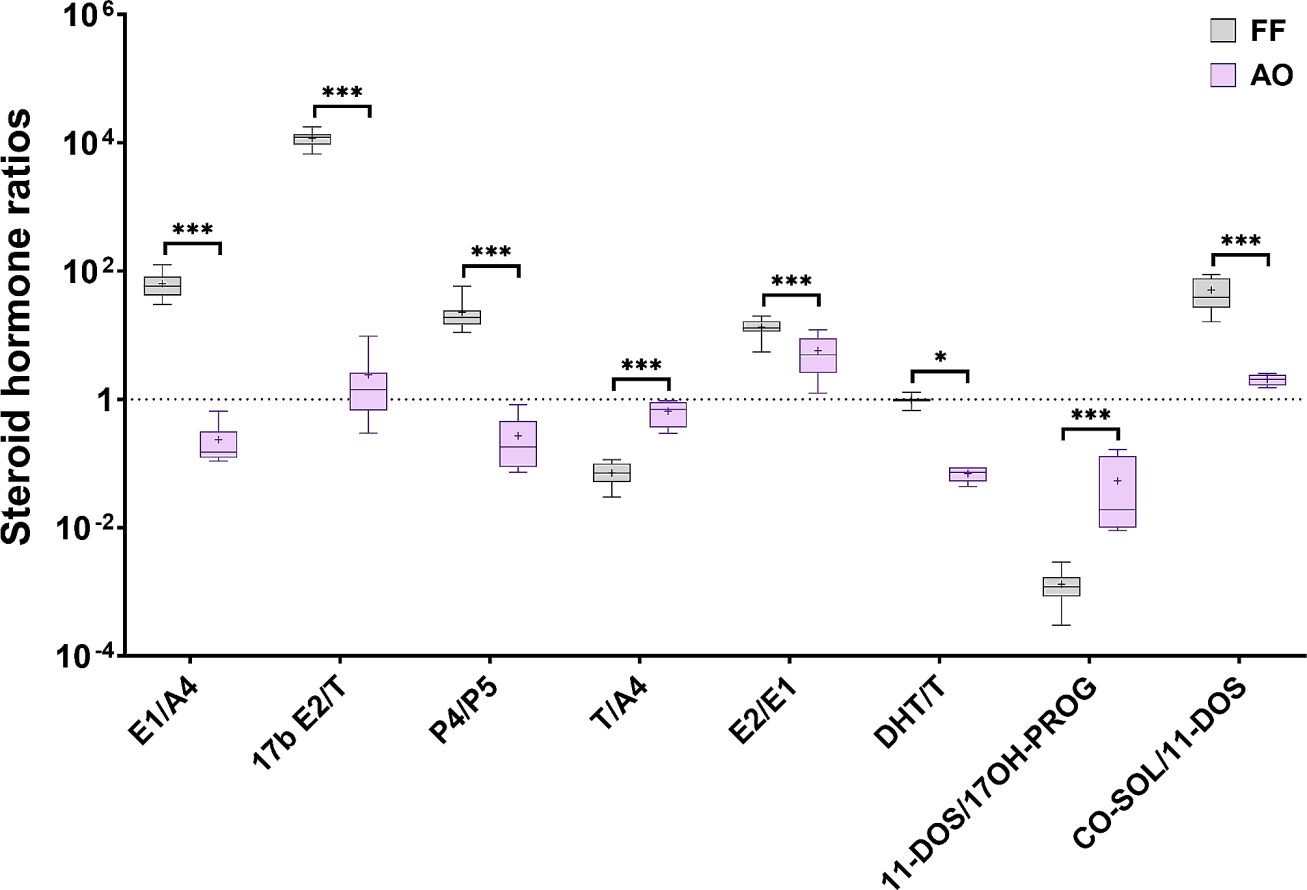
Ratios of steroid hormones from follicular fluid (FF) and adult ovarian tissue cultures (AO). Median ratios (middle line), 25% and 75% percentiles (box borders) are shown on a logarithmic scale. Mean ratios indicated as ”+” and whiskers indicate the minimum and maximum values. Statistically significant differences in steroid hormone rations between follicular fluid and adult ovarian cultures are shown by **(*p* < 0.01) and ***(*p* < 0.001)

Steroid hormone ratios were compared between adult ovarian tissue cultures and follicular fluid samples to reflect differences in steroidogenic enzymatic activity by early growing follicles in ovarian cortical tissue cultures from C-section patients and in follicular fluid of mature antral follicles during infertility treatments (Table S9; Table S12). Ratios for E1/A4, E2/T, P4/P5, E2/E1, DHT/T and CO-SOL/11-DOS were statistically significantly higher in follicular fluid samples than in adult ovarian tissue cultures (Fig. 6). In contrast, T/A4 and 11-DOS/17OH-PROG ratios were statistically significantly higher in adult ovarian tissue cultures (Fig. 6).

### Clustering of human ovarian-derived samples based on steroid hormone levels

**Fig. 7:**
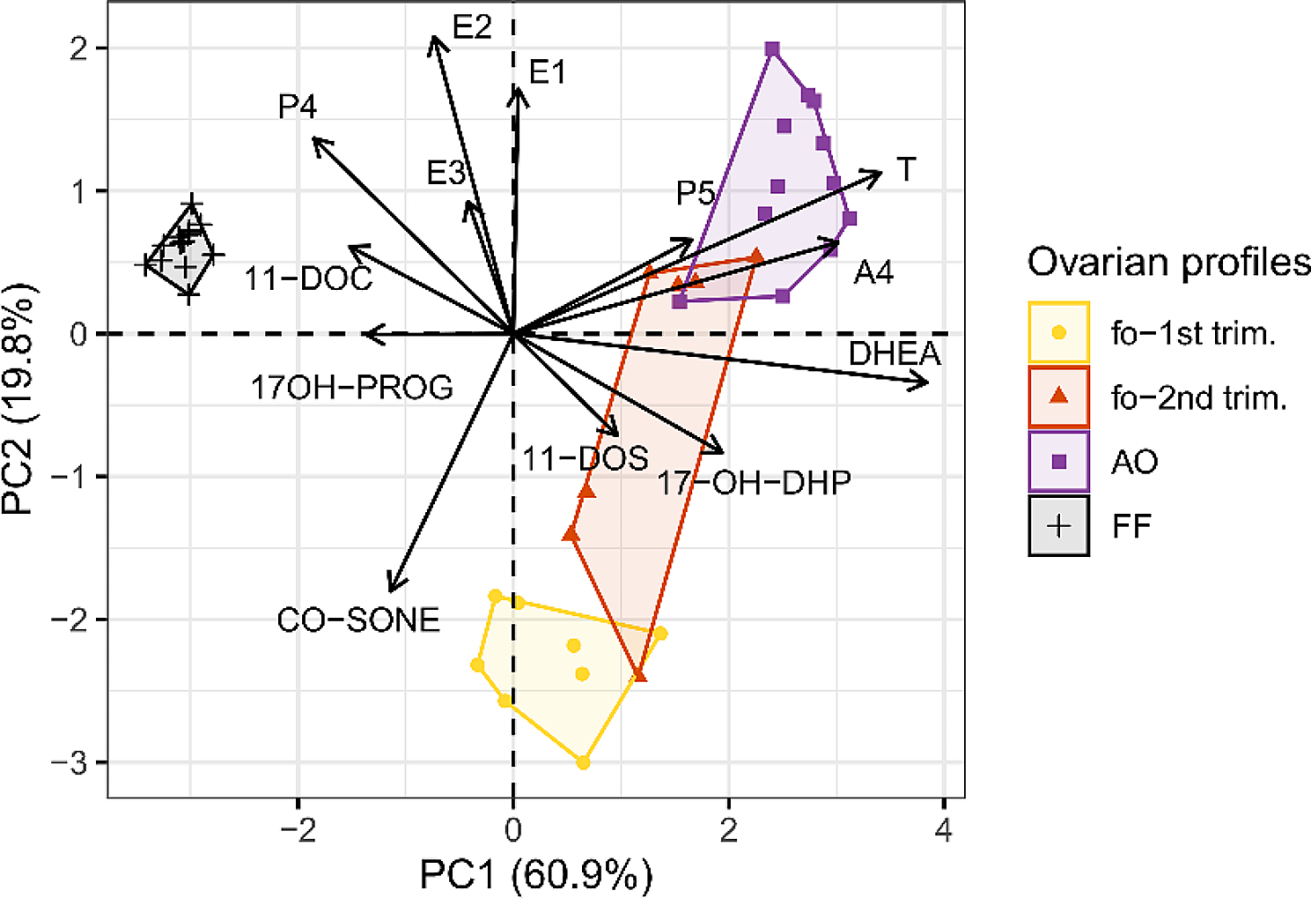
PCA biplot of steroid hormone profiles in fetal ovarian tissue cultures (fo), human adult ovarian tissue cultures (AO) and follicular fluid (FF). The steroid hormones that explained most of the total variance based on PC1 and PC2 and contributed most to the clustering are shown next to black arrows

Principal component analysis (PCA) was applied to compare the steroid hormone profiles among all the ovarian-derived sample types. Follicular fluid, adult ovarian cortex and first and second trimester fetal ovarian tissue cultures formed clusters based on PC1 and PC2 with 60.9% and 19.8% explained variability, respectively (Fig. 7). DHEA, T and A4 contributed to 54% of the explained variability for PC1 and PC2 with 21%, 19% and 14% contribution, respectively (Table S13). Ovarian tissue cultures from women and fetuses showed higher inter-individual variability in steroid hormone profiles, creating wider clusters than the follicular fluid samples. Clusters from adult ovarian tissue cultures mainly differentiated due to relatively more abundant P5, DHEA, T, and A4 compared to the other ovarian-derived sample types (Fig. 7). The second trimester fetal ovarian tissue cultures overlapped partially with the first trimester and adult ovarian tissue samples, but high abundance of DHEA, 11-DOS and 17-OH-DHP in the second trimester contributed to their partial separation. Follicular fluid samples formed a distinct cluster mainly due to relatively less abundant P5, DHEA, T and A4, but also higher abundance of P4 and E2 compared to the ovarian tissue culture samples (Table S13; Fig. 7).

## Discussion

We have developed an LC-MS/MS method with high recovery and low variability for the quantification of 21 steroid hormones. Using this method, we show clear differences in steroid hormone profiles among different human ovarian-derived sample types, including fetal ovarian tissue culture media, which on its own is challenging due to the small tissue sizes and low levels of hormones produced. Here, we show that fetal ovaries have the potential to synthesize progestogens, androgens, estrogens and, interestingly, corticosteroids. Since reproductive development and early-life programming of the hypothalamus-pituitary-adrenal axis is more sensitive to changes in cortisol levels in females than in males [23, 24], this stresses the importance of investigating possible production of corticosteroids in developing ovaries. In our study, second trimester fetal ovaries showed higher release of androgens and estrogens compared to the first trimester ovarian cultures. Lecante et al. found similar levels of release rates of progestogens P5, P4, 17OH-PROG, and E2 in fetal ovarian tissue cultures (GW 12 to 14) [10]. Increasing steroidogenic capacity is expected with advancing development of the female reproductive tract, as the expression of steroidogenic enzymes increases and primordial follicles start to form around 17 weeks of gestation [8]. However, the exact composition of endocrine cells present in fetal ovaries at various developmental stages requires further characterisation. In adult ovarian tissue cultures from C-section patients, steroid hormones are secreted by both theca and granulosa cells of primordial up to secondary pre-antral follicles [22]. Ovarian stroma produces only low levels of steroids in culture [25]. Hao et al. showed that the E2/T ratio correlates with secondary follicle counts in cultured ovarian cortex tissue from C-section patients, suggesting that cultured follicles actively produce estrogens [26]. In addition, they found ranges of P4, 17OH-PROG, DHEA, A4, T and E2 in their culture media that are comparable to our study, but did not detect DHT, CORTICO and CO-SOL, possibly due to their higher limits of detection. Another study reported E2 and P4 secretion by adult human ovarian cortical tissues from C-section patients at similar levels to our lowest reported levels [27]. Steroid production in fetal and adult ovarian tissue cultures typically shows high interindividual variability. While in present study steroid hormone release rates were calculated to reduce variablity due to sample size and culture time, this did not account for variations in the quantity and types of follicles present in the tissues. More spatially discriminating research is needed to delineate individual factors affecting steroid production by ovarian tissues in culture. Moreover, the synthesis of especially corticosteroids in the (developing) ovary warrants further attention.

The follicular fluid samples in this study were obtained from an IVF cohort and thus represent mature antral follicles from unnaturally stimulated cycles with high steroidogenic capacity [28]. Not surprisingly, progestogens (P4, 17OH-PROG) and estrogens (E2, E3) were more abundant in follicular fluid than in the adult and fetal ovarian tissue cultures. Similar ranges of steroid hormones in follicular fluid were found in a previous study [29]. In contrast, other studies showed similar P5, P4, 17OH-PROG, E1 and E2 levels, but 16–200 times higher T and A4 levels [30–32]. In addition, some studies using LC-MS/MS reported levels of DHEA, 17-OH-PREG and AN-DAN, which were not detected in the present study [29, 31, 32]. The variations in androgen levels especially might be explained by differences in IVF treatment dosing and timing, and inter-individual variability. Notably, our samples were retrieved from leading follicles only, while some other studies have pooled follicular fluids from all punctured follicles. Another explanation may be the differences in sample preparation and analytical methods. For example, cross-reactivity for androgens in immunoassays is especially prevalent in follicular fluid samples [29, 32, 33].

Interestingly, 17 A-E2 was found in all follicular fluid samples in our study. 17 A-E2 is a naturally occurring structural isomer of E2, but shows reduced activation of estrogen receptor alpha and beta, and has lower feminizing effects than E2 [34]. 17 A-E2 is considered a moderate 5-alpha reductase inhibitor [35]. It was suggested that 17 A-E2 may reduce the ovarian primordial follicle depletion, but this could not be confirmed in female mice [34]. Thus, the function of 17 A-E2 in ovaries in mice and humans remains elusive and warrants further studies into its physiological role.

Of the three backdoor steroid hormones determined, 17-OH-DHP was most prevalent and was found in 13/14 follicular fluid samples, 2/8 first and 2/7 second trimester fetal ovarian cultures, and 1/13 adult ovarian culture. A similar study with second trimester human fetal ovaries detected backdoor steroids 17-OH-DHP, 5α-17-hydroxypregnanolone and ANDROST, but not AND-AN in culture media [36]. A study with first trimester human female and male fetal gonad explant cultures demonstrated the formation of backdoor pathway intermediate steroids AND-AN, ANDROST, 5α-17-hydroxypregnanolone [37]. However, in that study, 17-OH-PROG and androgens were added to the culture media to simulate 21-hydroxylase deficiency and enhance formation of backdoor steroid hormones [37]. In the follicular fluid samples in our study, AND-AN was not detected but it has previously been detected in follicular fluid of regularly menstruating women and women undergoing IVF treatment [31]. More studies are clearly needed to elucidate the function and importance of backdoor pathway steroid hormone biosynthesis in the ovaries at different life stages.

Multiple enzymes are involved in steroidogenesis and include several cytochrome P450 (CYP) enzymes and hydroxysteroid dehydrogenases (HSDs) [38]. The steroid hormones measured in the present study may result from *de novo* synthesis by ovarian cells, by conversion of intracellularly retained steroid hormones from nongonadal sources, such as the placenta [36] or adrenals [39], or by conversion of steroid hormones present in culture media. Nevertheless, the detection of 11-DOS and 11-DOC in both fetal and adult ovarian tissue cultures suggest the capability of intra-ovarian corticosteroid biosynthesis. To our knowledge, this has not been described so far, though low expression of *CYP21* mRNA has been shown in second trimester fetal ovaries [40]. The CO-SONE detected in fetal ovarian cultures may reflect direct conversion of CO-SOL from the culture media by HSD11B2. Adult ovaries are known to express HSD11B1, and its expression is increased in granulosa cells after LH surge [41]. In fetal ovarian cultures, E1/A4 and E2/T ratios, indicative of CYP19A1 (aromatase) activity, were higher compared to adult ovarian cultures. Low aromatase activity in adult ovarian tissue cultures may have been due to the luteo-placental shift that occurs during pregnancy [42]. However, in many species, fetal ovarian aromatase expression and activity are considered to be extremely low [43–45]. Nevertheless, increasingly widespread expression of CYP19A1 occurs in human fetal ovaries between GW 12 and 14–16, and high expression in pre-granulosa cells of primordial follicles around GW 18–19 [8, 10]. Similarly, 17OH-PROG/P4 was significantly higher in fetal than in adult ovarian tissue cultures, suggesting high fetal CYP17A1-hydroxylase activity. Interestingly, CYP17 activity is considered a marker of theca cell activity in mature follicles in adult women. Nevertheless, some expression of *CYP17A1* mRNA occurs in second trimester fetal ovaries [40]. CYP17A1-positive immunostaining is visible from GW 14–16 onwards in some oocytes and a few somatic cells in human fetal ovaries [8]. In the current study, ovarian gene and protein expression of steroidogenic enzymes were not determined. Taken together, these data demonstrate the capacity of both fetal and adult ovaries to synthesize steroid hormones. PCA further supported the differing steroidogenic capacities of the analysed samples, clearly grouping the ovarian-derived samples by their origin. Further studies are needed to confirm the different steroidogenic capacities between fetal and adult ovarian tissues to better understand their role in ovarian physiology and the possible adverse effects of EDCs.

The major limitations of our study are the relatively small number of samples and high inter-individual variability, especially for the fetal and adult ovarian tissue cultures. In addition, the culture of ovarian tissues does not capture the involvement of the hypothalamic-pituitary-gonad/adrenal axes, which may affect the biological status of the ovarian tissues. To gain a better understanding of physiological changes in human ovaries, it would be relevant to study steroidogenic profiles in ovarian tissues and follicular fluids from non-pregnant women with regular menstrual cycles. However, these tissues are difficult to obtain without invasive techniques.

## Conclusions

Female reproduction is sensitive to endocrine disruption but current limited understanding of life stage-dependent differences in steroid hormone profiles, particularly in the human ovary, hampers good mechanistic studies. Here, the availability of primary human tissues and robust methods to quantify low levels of steroid hormones, are indispensable. We have demonstrated the capacity of fetal and adult ovarian tissues to synthesize a broad range of steroid hormones, including corticosteroids and backdoor pathway steroid hormones. Moreover, this study revealed clear differences in ovarian steroid hormone profiles at different lifestages. Altogether, these data contribute to an increased understanding of human ovarian steroidogenesis and may support studies to investigate the impact of endocrine disruption on female reproductive health.

### Electronic supplementary material

Below is the link to the electronic supplementary material.

Supplementary Material 1

Supplementary Material 2

## Data Availability

No datasets were generated or analysed during the current study.
